# Phylogenetic signal in gut microbial community rather than in rodent metabolic traits

**DOI:** 10.1093/nsr/nwad209

**Published:** 2023-07-28

**Authors:** Xue-Ying Zhang, Saeid Khakisahneh, Wei Liu, Xinyi Zhang, Weiwei Zhai, Jilong Cheng, John R Speakman, De-Hua Wang

**Affiliations:** State Key Laboratory of Integrated Management of Pest Insects and Rodents, Institute of Zoology, Chinese Academy of Sciences, Beijing 100101, China; State Key Laboratory of Integrated Management of Pest Insects and Rodents, Institute of Zoology, Chinese Academy of Sciences, Beijing 100101, China; State Key Laboratory of Integrated Management of Pest Insects and Rodents, Institute of Zoology, Chinese Academy of Sciences, Beijing 100101, China; Key Laboratory of Zoological Systematics and Evolution, Institute of Zoology, Chinese Academy of Sciences, Beijing 100101, China; CAS Center for Excellence in Animal Evolution and Genetics, University of Chinese Academy of Sciences, Kunming 650223, China; Key Laboratory of Zoological Systematics and Evolution, Institute of Zoology, Chinese Academy of Sciences, Beijing 100101, China; CAS Center for Excellence in Animal Evolution and Genetics, University of Chinese Academy of Sciences, Kunming 650223, China; Key Laboratory of Zoological Systematics and Evolution, Institute of Zoology, Chinese Academy of Sciences, Beijing 100101, China; Shenzhen Key Laboratory of Metabolic Health, Center for Energy Metabolism and Reproduction, Shenzhen Institutes of Advanced Technology, Chinese Academy of Sciences, Shenzhen 518055, China; Institute of Biological and Environmental Sciences, University of Aberdeen, Aberdeen AB39 2PN, UK; State Key Laboratory of Molecular Developmental Biology, Institute of Genetics and Developmental Biology, Chinese Academy of Sciences, Beijing 100101, China; State Key Laboratory of Integrated Management of Pest Insects and Rodents, Institute of Zoology, Chinese Academy of Sciences, Beijing 100101, China; School of Life Sciences, Shandong University, Qingdao 266237, China; CAS Center for Excellence in Biotic Interactions, University of Chinese Academy of Sciences, Beijing 100049, China

**Keywords:** gut microbiota, metabolic plasticity, phylogenetic signal, host–bacteria interactions, air temperature (*T*_a_)

## Abstract

Host phylogeny and environment have all been implicated in shaping the gut microbiota and host metabolic traits of mammals. However, few studies have evaluated phylogeny-associated microbial assembly and host metabolic plasticity concurrently, and their relationships on both short-term and evolutionary timescales. We report that the branching order of a gut microbial dendrogram was nearly congruent with phylogenetic relationships of seven rodent species, and this pattern of phylosymbiosis was intact after diverse laboratory manipulations. Laboratory rearing, diet or air temperature (*T*_a_) acclimation induced alterations in gut microbial communities, but could not override host phylogeny in shaping microbial community assembly. A simulative heatwave reduced core microbiota diversity by 26% in these species, and led to an unmatched relationship between the microbiota and host metabolic phenotypes in desert species. Moreover, the similarity of metabolic traits across species at different Tas was not correlated with phylogenetic distance. These data demonstrated that the gut microbial assembly showed strong concordance with host phylogeny and may be shaped by environmental variables, whereas host metabolic traits did not seem to be linked with phylogeny.

## INTRODUCTION

Mammals rely on regulating metabolic rates to keep their core body temperature relatively stable in diverse environments. As supposed by the classical metabolic theory of ecology [1,2], the metabolic rate is proportional to body mass with an allometric relationship, and is affected particularly by air temperature (*T*_a_). Although the diversity of body sizes and metabolic rates are primarily determined by genetics [3], mammals display metabolic plasticity and are subjected to thermal constraints in response to varying environments [4,5]. The plasticity of *T*_a_-induced metabolic rates has been suggested to be a key factor determining population extinction or survival in a warming world [6]. Therefore, elucidating the complex evolutionary mechanisms of metabolic phenotypes that allow mammals to accommodate such a variable, wide range of thermal environments is of importance for understanding biodiversity distribution and predicting responses to the warming climate, particularly responses to extreme *T*_a_s (such as during heatwaves).

Increasing evidence indicates that the metabolic traits of mammals are the consequence of interaction between gut microbiota and their hosts [7]. Recently, a pattern of phylosymbiosis, showing that gut microbial communities might reflect the phylogeny of related host species, has been documented in different animal groups with distant phylogenetic relationships [8,9], and thus closely related species may harbor similar microbes [10–12]. One possible mechanism that may explain the phylogenetic relationship is the vertical transmission of original microbiota from the mother and relatives with close contact across generations [13,14]. However, the transmission may be unfaithful, possibly due to host filtering [15–18]. Besides vertical transmission, some other mechanisms are also able to explain covariation between hosts’ phylogeny and characteristics of their gut microbiota. Importantly, most (intraspecific) variations in gut microbiota are explained by environmental factors, such as diet, *T*_a_ and social interaction [19–21]. Gut microbiota can be transmitted horizontally between individuals or from the environment [22]. Alterations in the gut microbiota composition are accompanied by changes in bacterial metabolites and neurotransmitters, which may modulate host physiology and behavior [23,24]. Therefore, it is generally proposed that host–microbe associations contribute to maximizing a host's fitness in variable environments.

Some studies in human and animal models with broad phylogenetic clades suggest a genetic determinant of gut microbiomes in natural conditions [11,25–27], whereas others did not find such evidence [28,29]. Besides, environment-induced variations in gut microbiota determine host phenotypes and fitness, despite evidence that host metabolic variation is the consequence of genetic variations [3]. Therefore, the relative contributions of host phylogeny (genetics) and environmental variables in affecting gut microbial assembly and host metabolic plasticity are still controversial. Moreover, studies evaluating functional significance and physiological mechanisms were generally focused on laboratory animal models or only on a single wild species, and therefore it is almost impossible to predict the general or specific patterns of responses in gut microbiota and host metabolic plasticity to changing environments without interspecies comparisons. More importantly, few studies have evaluated phylogeny-related microbial assembly and host metabolic traits concurrently, and their relationships on short-term and evolutionary timescales.

Here, we address these questions and elucidate the relative contributions of host phylogeny and different environments (such as laboratory rearing, diet and *T*_a_) on the gut microbial community and host metabolic traits in several rodent species. We demonstrate that gut microbial communities are distinguishable by host phylogeny (clades) under different environments, and the microbial responses to high *T_a_* were phylogenetically conserved so that closely related rodent taxa and bacterial taxa responded more similarly than those related distantly. In contrast, host metabolic plasticity in response to variation in *T_a_* was more related to environment, rather than phylogeny. Moreover, heat-induced reductions in microbial diversity and alterations in microbial composition were paralleled with decreases in host metabolic traits in most species. The desert rodents, however, exhibited decoupled variations between gut microbiota and metabolic traits in response to heatwaves.

## RESULTS

### Phylogeny overrides environments in driving gut microbiota structure

**Figure 1.**
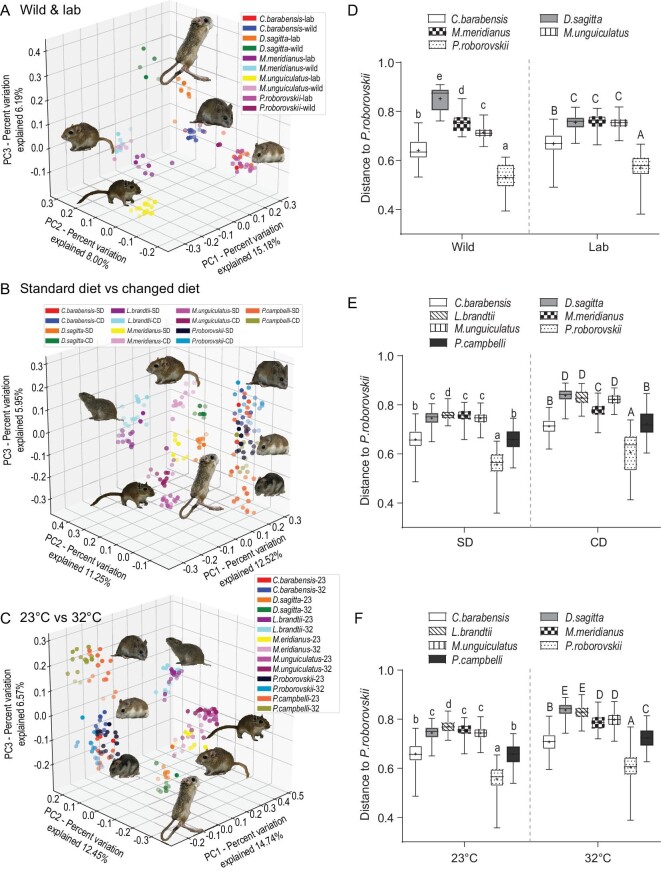
Microbial community in fecal samples across rodent species, between wild and laboratory-reared populations, with different diets or at different temperatures. (A) Principal coordinate analysis (PCoA) plots of Bray-Curtis distance matrix in fecal microbiota colored by wild and lab for different species. (B) PCoA plots of Bray-Curtis distance matrix in fecal microbiota colored by species and diet (standard diet, SD; changed diet, CD). (C) PCoA plots of Bray-Curtis distance matrix in fecal microbiota colored by species and air temperature (23^o^C, 23; 32^o^C, 32). (D–F) Comparisons of Bray-Curtis distances of species to *P. roborovskii* between wild and laboratory-reared populations, with different diets (SD and CD) or at different temperatures (23^o^C and 32^o^C). Different small and capital letters above box plots indicate significant differences between species in the wild and lab, or with SD and CD, or at 23^o^C and 32^o^C, respectively (*P* < 0.05).

To compare interspecies differences in the gut microbiota of rodent species and distinguish the contribution of phylogeny and environmental factors (wild vs. captive, diet and *T_a_*) in driving the divergence of gut microbial communities, we analyzed 16S rRNA gene amplicon sequencing in seven rodent species (*Cricetulus barabensis, Dipus sagitta, Lasiopodomys brandtii, Meriones meridianus, Meriones unguiculatus, Phodopus roborovskii* and *Phodopus campbelli*) from three families (Cricetidae, Dipodidae and Muridae), which are representative species of the grasslands of Inner Mongolia, China. These species were from wild or captured populations, and the captured rodents were acclimated to a standard diet (SD, rabbit pellet chow with 12.4% fiber content for *L. brandtii*, and rat pellet chow with 3.5% fiber content for other species based on their diet habits in the wild) or changed diets (CD, exchanged the diets), and were acclimated to 23^o^C (room temperature) or 32^o^C (simulated heatwaves, at the upper edge of the thermoneutral zones of all species). The community dissimilarities (β-diversity) indicated by principal coordinate analyses (PCoA) based on Bray-Curtis distance showed that gut microbial communities were distinguished by species in both wild and captured populations (Fig. 1A–C). Specifically, the microbial communities for laboratory-reared animals were separated into three clusters by species (Analysis of similarities, ANOSIM, *R* = 0.970, *P* = 0.001, Fig. 1B, C). One cluster was for *L. brandtii* (herbivore), which harbored microbial communities dramatically different from all other species. The second was for *C. barabensis, P. roborovskii* and *P. campbelli* (omnivore), which shared some similar microbial communities but differed from other species. Another cluster was for the microbial communities from *D. sagitta, M. meridianus* and *M. unguiculatus* (granivore), which were much closer but also exhibited some small separation between species. The changes in diet (ANOSIM, *R* = 0.164, *P* = 0.001) or *T_a_* (ANOSIM, *R* = 0.056, *P* = 0.001) also led to a smaller separation of microbial communities (Fig. 1B, C). The distance matrix showed that *P. roborovskii* was much closer to *P. campbelli* and *C. barabensis*, next closer to *M. meridianus, M. unguiculatus* and *D. sagitta*, and then closer to *L. brandtii* independent of diet or *T_a_* (Fig. 1D–F). This suggests that gut microbiota community is distinguished by host species.

### A gut microbial dendrogram reflects host phylogenic signal independent of the environment

**Figure 2.**
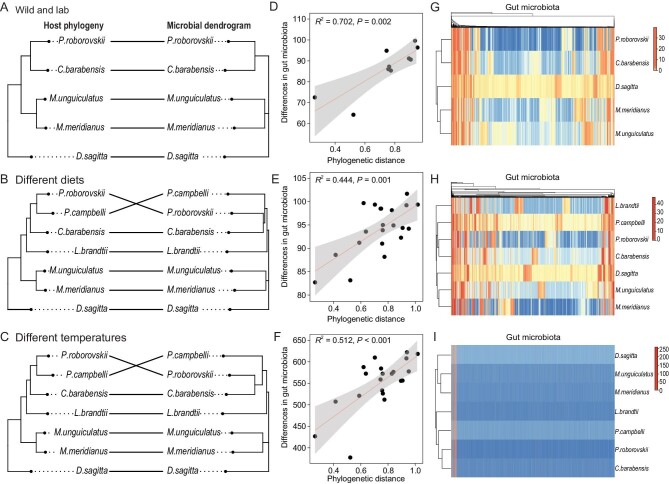
The relationships between host phylogeny and gut microbial dendrogram in different conditions. (A–C) The host species phylogenetic tree (left) was based on concatenate genes from the Bayesian inference analysis. The gut microbial dendrogram (right) was based on the similarities in the pooled bacterial communities of each host species. The microbial dendrogram mirrored the host phylogenetic relationship (A, RF distance = 0, *P* = 0.077; B and C, RF distance = 2, *P* = 0.009). (D–F) The linear regression analysis showed that the differences in the gut microbiota between species were positively correlated with host phylogenetic distance at different conditions. The lines represent the trend lines which were created using linear models, and the gray shadows indicate 95% confidence intervals. Mantel tests based on Spearman's correlation also supported a significant phylogenetic signal of β diversity distances (wild vs. lab, *r* = 0.746, *P* = 0.05; diet, *r* = 0.466, *P* = 0.032; *T*_a_, *r* = 0.555, *P* = 0.026). (G–I) The heat maps reflect the similarities in the pooled bacterial communities of each host species. RF, Robinson-Foulds.

We then evaluated whether diversity in the gut microbiota across rodent species in different conditions was driven by genetic variations of host species (phylogeny) rather than environment. The phylogenetic tree of these rodents was reconstructed based on the mitochondrial DNA (mtDNA) of cytochrome b (*Cytb*) and cytochrome oxidase subunit I (*COI*), and the nuclear DNA (nDNA) of interphotoreceptor retinoid-binding protein (*IRBP*). The phylogenetic topologies showed that *D. sagitta* was monophyletic and placed as a sister to all other species (Fig. 2A–C, left). The two species *M. meridianus* and *M. unguiculatus* from Gerbillinae were clustered and were sisters to species from Cricetinae. The phylogeny within Cricetinae contained four species. *P. roborovskii* and *P. campbelli* were clustered and were sister species to *C. barabensis. L. brandtii* was the most distinct from other Cricetinae species. The microbial dendrogram (Fig. 2A–C, right) was built based on the similarity (Euclidean distance) of the bacterial communities of each host. Based on the calculation, using phytools, of the Robinson-Foulds (RF) distance and permutations to assess the significance, we observed that the two trees were identical (RF distance = 0, *P* = 0.077, Fig. 2A), and there were two partitions of data present in one of the trees but not in the other (RF distance = 2, *P* = 0.009, Fig. 2B, C). These data indicate that host species from the same branching genus shared similar microbial communities and only the microbial similarity between *C. barabensis* and *P. campbelli* did not match with their hosts’ phylogenetic relationships (Fig. 2A–C). The distances in gut microbiota between species were positively correlated with host phylogenetic distance in different rearing conditions (*R*^2^ = 0.702, *P* < 0.01; Fig. 2D), under different diets (*R*^2^ = 0.444, *P* < 0.001; Fig. 2E) or at different *T*_a_s (*R*^2^ = 0.512, *P* < 0.001; Fig. 2F). Mantel tests based on Spearman's correlation also supported a significant phylogenetic signal of β diversity distances (wild vs. lab, *r* = 0.746, *P* = 0.05; diet, *r* = 0.466, *P* = 0.032; *T*_a_, *r* = 0.555, *P* = 0.026). These relationships between host species and their microbes are displayed via heat maps (Fig. 2G–I). All these comparisons indicate that the microbial dendrogram reflects host phylogenetic signal as being relatively invariant with respect to the environment.

**Figure 3.**
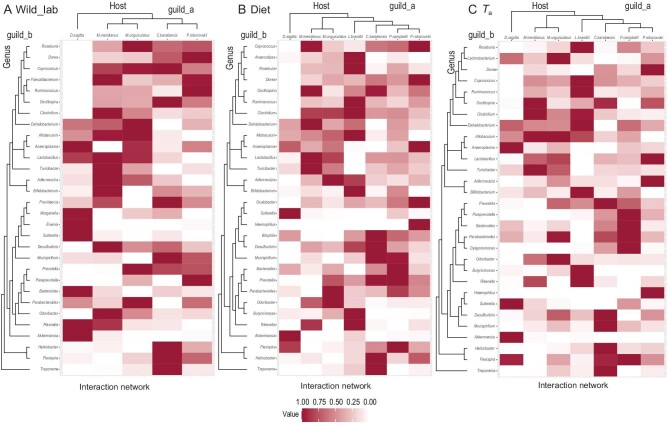
Illustration of the testing of the phylogenetic signal with an interaction network between hosts and the genus of bacteria, with associated phylogenetic trees. The bipartite interaction networks between hosts (guild_a) and the top 30 genera of bacteria (guild_b) under (A) wild and lab conditions, (B) different diet and (C) temperature (*T*_a_) treatments are represented by a matrix, indicating the interaction extent using shades from light (no interaction) to dark (many interactions). These guilds are characterized by the rooted phylogenetic trees of rodent hosts and top 30 genera of gut bacteria, respectively, which were used to calculate the phylogenetic distances between pairs.

We further used the phylosignal_network function of R-package RPANDA and did Step 1 and Step 2 to evaluate a phylogenetic signal in host–bacteria interactions when accounting for the signal in the number of partners [30,31]. We observed that closely related rodent species interacted with similar bacteria (weighted, in Step 1 pvalue_upper_a < 0.05, Table S1, Fig. 3), and similarly, closely related bacteria species interacted with similar rodent species (weighted, in Step 1 pvalue_upper_b < 0.05, Table S1). Moreover, the significant phylogenetic signal in species interactions between rodents and their gut microbiota cannot be fully explained by the phylogenetic signal in the number of partners (weighted, in Step 2 pvalue_upper_a < 0.05). When the relative abundance of bacteria species was not considered however (unweighted, in Step 1 pvalue_upper_a < 0.05 and in Step 2 pvalue_upper_a > 0.05), we cannot rule out the possibility that the phylogenetic signal in host–bacteria interactions observed in Step 1 was not explained by the phylogenetic signal in the number of partners. Therefore, the data imply that signals come from the species identity and not only the number of partners in host–bacteria interactions when both bacteria species and their relative abundance were considered.

### A simulated heatwave leads to a large reduction in core microbiota

**Figure 4.**
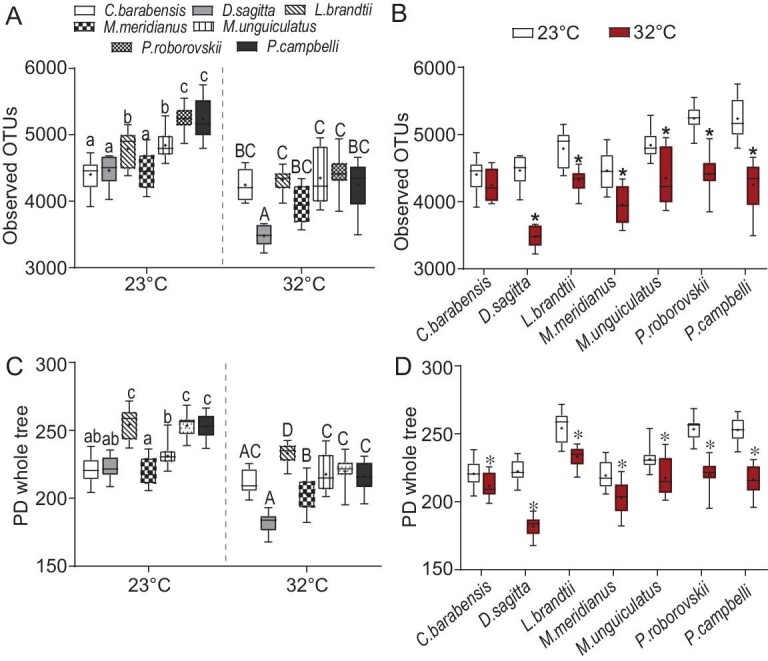
Variations in the α diversity of microbial communities in fecal samples of seven rodent species in response to high *T_a_*. (A, B) The number of OTUs observed in the samples. (C, D) The analysis of phylogenetic diversity (PD) whole tree in the samples. ‘+’ indicates the mean of the data. Different small and capital letters above box plots indicate significant differences between species at 23^o^C and 32^o^C, respectively (*P* < 0.05). *P < 0.05, 32^o^C vs. 23^o^C.

Climate warming and/or frequently occurring heatwaves have been found to reduce the diversity of microbial communities and decrease fitness of ectotherms [32,33]. However, how the gut microbiota of endotherms respond to heatwaves has been rarely reported. We next tested changes of the gut microbiota in laboratory-reared rodent species in response to high *T_a_* (a mimicked heatwave). The animals were acclimated either at 23ºC (room temperature) or at 32ºC for 3 weeks. Using phylogenetic generalized linear models (PGLMs), we observed no phylogenetic relationships and there were significant differences in the α diversity of microbial communities between rodent species (Lambda = 0, *P* < 0.001; Table S2). The observed operation taxonomic units (OTUs) in *P. roborovskii* and *P. campbelli* showed the highest level, and those in *C. barabensis, D. sagitta* and *M. meridianus* showed the lowest at 23ºC (*P* < 0.001; Fig. 4A, B). High *T_a_* led to a decrease in the OTUs in all species except *C. barabensis*, and larger reductions in observed OTUs such as in *D. sagitta* (by 22.1%) and *P. roborovskii* (by 18.8%) (Fig. 4B). Both at 23ºC (*F*_6, 81_ = 37.013, *P* < 0.001) and 32ºC (*F*_6, 80_ = 25.595, *P* < 0.001), *L. brandtii, P. roborovskii* and *P. campbelli* had higher index of phylogenetic diversity (PD) whole tree in their microbial communities than other species, and the index of PD whole tree in *M. unguiculatus* was higher than *M. meridianus* (Fig. 4C, D). High *T_a_* led to a dramatic decrease, in all the parameters (Chao1, observed OTUs, Shannon and Simpson index, and PD whole tree), of the α diversity of gut microbial communities (*P* < 0.001; Table S3). The core microbes shared by 90% of samples in the groups were defined as the active OTUs. The core microbe analysis at 23^o^C identified 288 common OTUs observed in all 7 species, 42 common OTUs observed in both *P. roborovskii* and *P. campbelli*, 22 common OTUs observed in both *M. meridianus* and *M. unguiculatus*, and 497 specific core OTUs in *L. brandtii* (Fig. S1A). High *T_a_* led to a 26% decrease in the core OTUs of all species (Fig. S1B).

The differential bacterial taxonomy was identified by the linear discriminant analysis (LDA) Effect Size (LEfSe) method for different rodent species (LDA score > 3, Fig. S2A, B) and at different *T_a_*s (Fig. S2C, D). A total of 14 different bacterial phyla (mean relative abundance > 1%) were observed in these 7 rodents. The dominating bacterial phyla included Firmicutes, Bacteroidetes, Proteobacteria, Tenericutes and Actinobacteria, and all these dominating phyla showed significant differences between species, both at 23ºC (except for Tenericutes) and 32ºC (Figs S3, S4). The Verrucomicrobia phylum was specifically observed in *D. sagitta* (3.50% at 23ºC and 5.90% at 32ºC), rather than in other species. At the genus and species levels, the relative abundance of specific bacteria exhibited species-specific differences (Fig. S5). The abundance of the *Dorea* genus was higher in *C. barabensis, P. roborovskii* and *P. campbelli* (Fig. S5A), and the *Flexispira* genus was also richer in these rodent species as well as in *D. sagitta* (Fig. S5B). *Lactobacillus* and *Odoribacter* were higher in *M. meridianus* and *M. unguiculatus* than in other species (Fig. S5C, D)*. L. brandtii* exhibited the highest abundance of the *Ruminococcus* genus, *Ruminococcus flavefactiens* species (Fig. S5E), and *D. sagitta* exhibited the highest abundance of the *Akkermansia* genus, *Akkermansia muciniphila* species (Fig. S5F). The gut microbes also exhibited diverse responses to 32ºC, and some pathogenic bacteria such as from the phylum Proteobacteria flourished at high *T_a_* (Table S4). These data indicate that heatwaves reduce diversity and disturb the structure of gut microbial communities, and some genera from the same family or even the same phylum show similar fluctuations in response to high *T_a_*.

### High *T_a_* leads to decreases in metabolic traits in a species-specific manner

**Figure 5.**
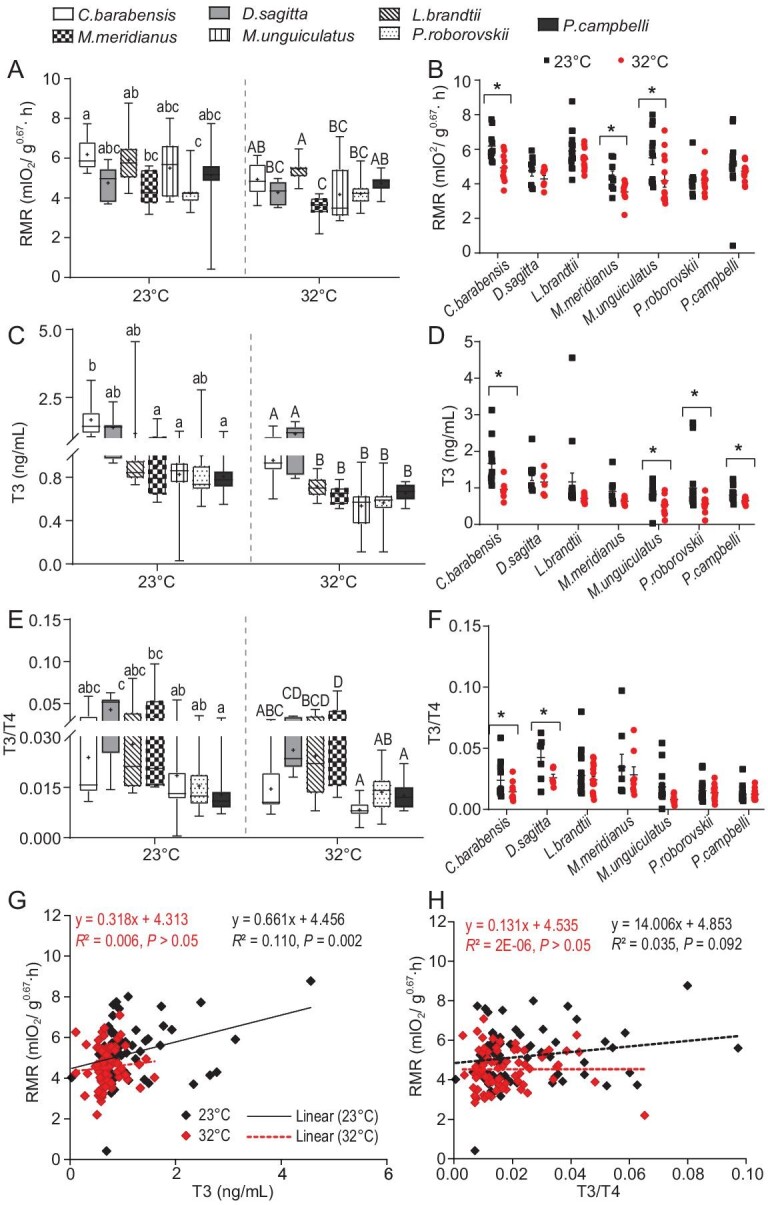
Resting metabolic rate (RMR) and serum thyroid hormones in seven rodent species at 23^o^C and 32^o^C. (A, B) RMR. (C, D) Serum T3 levels. (E, F) T3/T4 ratio. (G, H) The correlation between RMR and 558 serum T3 levels or T3/T4 ratio. Different small and capital letters above box plots indicate significant differences between species at 23^o^C and 32^o^C, respectively (*P* < 0.05). *P < 0.05, 32^o^C vs. 23^o^C.

We also examined the responses of metabolic traits in laboratory-reared rodent species acclimated to high *T_a_* (a mimicked heatwave). All metabolic traits showed no phylogenetic relationship and there were significant differences between species (PGLMs, Lambda = 0, *P* < 0.05; Table S5). At both 23ºC and 32ºC, these seven species showed significant differences in body-mass-corrected resting metabolic rate (RMR, mlO_2_/g^0.67^·h, *P* < 0.001; Fig. 5A). *C. barabensis* and *L. brandtii* had higher RMR than *M. meridianus* and *P. roborovskii* (*P* < 0.05). After acclimation to 32ºC, RMR decreased by 8.6%–24% in most species (*T_a_, F*_1, 75_ = 21.679, *P* < 0.001; species, *F*_6, 75_ = 7.222, *P* < 0.001; Fig. 5A) and marked decreases were observed in *C. barabensis* (by 20.4%, *P* < 0.001), *M. meridianus* (by 20.4%, *P* = 0.010) and *M. unguiculatus* (by 24.0%, *P* = 0.009), but remained very stable in *P. roborovskii* (Fig. 5B). Serum levels of active 3,5,3’-tri-iodothyronine (T3), playing a key role in metabolic regulation, showed significant differences between species at both 23ºC (*F*_6, 88_ = 3.315, *P* = 0.006) and 32ºC (*F*_6, 82_ = 14.959, *P* < 0.001; Fig. 5C). Serum T3 levels in *C. barabensis* were higher than those in *M. meridianus, M. unguiculatus* and *P. campbelli* at 23ºC, and after being acclimated to 32ºC, both *C. barabensis* and *D. sagitta* showed higher serum T3 levels compared to other species (Fig. 5D). All the species reduced serum T3 levels in response to 32ºC acclimation (*T_a_, F*_1, 70_ = 25.414, *P* < 0.001; species, *F*_6, 70_ = 6.069, *P* < 0.001) and significant differences were observed in *C. barabensis* (by 42.8%, *P* = 0.016), *M. unguiculatus* (by 35.2%, *P* = 0.007), *P. roborovskii* (by 43.7%, *P* = 0.026) and *P. campbelli* (by 18.9%, *P* = 0.005). The ratio of T3 to inactive thyroxine (T4) also showed differences between species at both 23ºC (*F*_6, 88_ = 4.966, *P* < 0.001; Fig. 5E) and 32ºC (*F*_6, 82_ = 8.503, *P* < 0.001; Fig. 5F), and a significant reduction was observed in *C. barabensis* and *D. sagitta* at 32ºC compared to 23ºC (*P* < 0.05). Pearson correlation showed that serum T3 levels were positively correlated with RMR at 23ºC (*R*^2^ = 0.110, *P* = 0.002; Fig. 5G), but not at 32ºC (*R*^2^ = 0.035, *P* = 0.092). There is no correlation between the ratio of T3/T4 and RMR at both *T*_a_s (*P* > 0.05; Fig. 5H).

Pearson correlation analyses were performed to check the relationship between gut microbes and host metabolic traits. Significant correlations were identified between many specific OTUs and body mass, and only *Coprococcus* was observed to correlate positively with RMR in all rodent species at 23^o^C (Fig. S6A). In contrast, so many taxa correlated positively (such as *A. muciniphila* and *R. flavefactiens*) or negatively (*Dorea*) with body mass, RMR and serum T3 levels at high *T_a_* (Fig. S6B).

### Metabolic traits are related to *T*_a_ rather than phylogeny

**Figure 6.**
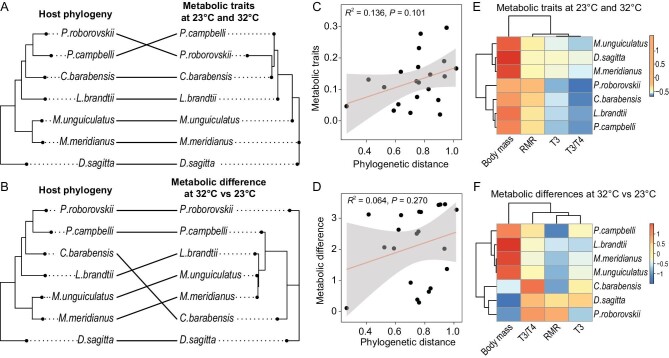
The relationship between phylogeny and metabolic traits or metabolic differences of rodent species at different *T_a_*s. (A, B) The host species phylogenetic tree (left) was based on concatenate genes from the Bayesian inference analysis, and the similarities of metabolic traits (the measured values of body mass, RMR and serum T3 and T3/T4 levels at both 23^o^C and 32^o^C) or metabolic differences (values at 32^o^C minus those at 23^o^C) were based on the pooled metabolic phenotypes of each species (right). The metabolic similarity did not match species phylogenetic relationships (A, RF distance = 4, *P* = 0.099; B, RF distance = 6, *P* = 0.232). (C, D) The linear regression analysis showed that the similarity in metabolic traits (*R*^2^ = 0.136, *P* = 0.101) or metabolic differences (*R*^2^ = 0.064, *P* = 0.270) between species was not significantly correlated with host phylogenetic distance. The lines are trend lines, which were created using linear models, and the gray shadows indicate 95% confidence intervals. (E, F) The heat maps reflect the similarities in the pooled metabolic traits or metabolic differences of each species. RMR, resting metabolic rate. RF, Robinson-Foulds.

To check whether variations in host metabolic traits at different *T_a_*s among rodent species are determined by phylogeny or environment, we further analyzed the relationship between the species phylogenetic tree and the similarity of metabolic phenotypes in responses to *T_a_* acclimation. The host species phylogenetic tree (Fig. 6A, B, left) was based on concatenate genes from the Bayesian inference analysis, and the similarities of metabolic traits (the measured values at both 23^o^C and 32^o^C, Fig. 6A, right) or metabolic differences (values at 32^o^C minus those at 23^o^C, Fig. 6B, right) were based on the pooled metabolic phenotypes of each species. The metabolic similarity did not match species phylogenetic relationships (RF distance = 4, *P* = 0.099, Fig. 6A; RF distance = 6, *P* = 0.232, Fig. 6B). The linear regression analysis showed that the similarity in metabolic traits (*R*^2^ = 0.136, *P* = 0.101) or metabolic differences (*R*^2^ = 0.064, *P* = 0.270) between species were not significantly correlated with host phylogenetic distance (Fig. 6C, D). Moreover, Mantel tests based on Pearson's correlation supported the idea that the dendrogram of metabolic traits (*r* = 0.368, *P* = 0.082; Fig. 6C) or metabolic differences (*r* = 0.252, *P* = 0.130; Fig. 6D) showed no phylogenetic signal. The species relationships of the metabolic traits (Fig. 6E) and metabolic differences across species at different *T*_a_s (Fig. 6F) are also displayed via heat maps. These data suggest that the phenotypic plasticity of the rodent species did not seem to be linked with phylogeny.

## DISCUSSION

Host phylogeny and environment have been implicated in shaping the gut microbiota and metabolic traits of mammals. In the present study, we compared the contributions of host phylogeny and environment to the gut microbial community, and also explored the association between gut microbiota and host metabolic adaptation to high *T_a_* in rodent species, including three families of Cricetidae, Dipodidae and Muridae. As predicted, we observed that more closely related rodent taxa shared similar gut microbiota. More importantly, this phenomenon still existed even under laboratory-reared, different-diet or *T*_a_ conditions. In addition, high *T*_a_ led to reductions in both gut microbial diversity and host metabolic traits. Core microbes showed a 26% reduction in all species, and these changes in the microbiota were correlated to host RMR (8.6%–24% lower at 32^o^C vs. 23^o^C). Moreover, metabolic traits were more related to *T*_a_ than the rodent's phylogeny. These data demonstrate a pattern of phylosymbiosis; the gut microbiota structures showed strong concordance with host phylogeny. In contrast, the phenotypic plasticity of the rodent species did not seem to be linked with phylogeny.

Increasing evidence has indicated that gut microbial communities might follow host phylogenetic relationships in different animal groups [8,11]. We observed that the branching orders of host, based on similarity of microbiota structure, were nearly congruent with the phylogenetic relationship of rodent species, suggesting a phylogenetic signal of the gut microbial community. A recent study in wild baboons revealed that microbiome heritability is common in some mammal lineages [25]. The data from small mammals, such as in the genera *Apodemus, Microtus* and *Sorex*, demonstrated that species genetics overrode environment in shaping the gut microbial community [34]. However, a study in humans showed that host genotype imposed a minor effect on gut microbiome composition [29], and the microbiomes of myrmecophagous mammals were also independent of the host phylogenetic relationship [35]. Mammal species may acquire and develop their gut microbial communities via vertical transmission through host generations [13,14]. There is less evidence for microbial vertical transmission between the Hawaiian *Ariamnes* spiders and their microbiota to explain the phylogenetic conservatism [36]. The present study supports the theory that a divergence of gut microbiota structures preserves the host phylogenetic signal. In contrast to previous studies, the present work not only compared the gut microbial communities of different rodent species between wild and laboratory conditions, but also tested this relationship by manipulating environmental variables, finding that the phylogenetic signal was intact after these diverse manipulations. Additionally, the phylogenic relationship of microbial communities within different families from one order (Rodentia) was tested, rather than from more divergent clades, therefore, it was possible to more accurately distinguish the critical role of phylogeny compared to environment.

Although significant vertical transmission and genetic components have been described for the gut microbiota assembly in mammals [25–27], most (intraspecific) variations are explained by environmental factors. Particularly, diets drive the convergence in gut microbial communities over short-term and evolutionary timescales [37,38]. In humans [28] and *Mus musculus domesticus* [39], the caecal bacterial diversity was strongly impacted by diets or geographical origins rather than their phylogenic relationships. Sympatric species such as the plateau pika (*Ochotona curzoniae*) and yak (*Bos grunniens*) exhibited convergence of microbiota due to horizontal transmission, which was a potential consequence of pikas eating yak feces [40]. The present study also implied the role of diet habits (omnivore, granivore and herbivore) or diet acclimation in shaping gut microbial communities. With regard to *T*_a_, the ectotherms are expected to be especially vulnerable to a global warming scenario. Climate warming induced a 34% loss in microbial diversity in *Zootoca vivipara* [32], and the reductions in gut microbiota through manipulation of environmental water sterilization were associated with the weakened thermal tolerance of hosts in response to both heat and cold stresses in tadpoles from *Lithobates clamitans* [33]. However, how endothermic mammals modulate their gut microbial community in response to heatwaves remains unknown. We observed a 26% reduction in core microbial diversity in these rodent species in the face of mimicked heatwaves. Moreover, the gut microbiota community of desert species is more sensitive and more easily disturbed by heat exposure. In comparison to the host genome, the gut microbiome might be more sensitive and vary more rapidly in response to an environmental disturbance and therefore may play a fundamental role in the processes of acclimation, adaptation and evolution of the holobiont (hosts and their gut microbiota).

Metabolic rate might have been strongly selected for by historically low *T*_a_ in order for small mammals to maintain euthermia in the cold. However, metabolic tolerance to high *T*_a_ (such as heatwaves) might have evolved more slowly [41]. High *T_a_* has been observed to lower RMR and survival [42], and also reduce reproductive performance in mice and *C. barabensis* [43]. Microbiota reduction was associated with impaired metabolic plasticity under both high and low *T_a_*s in mice, *L. brandtii* and *M. unguiculatus* [21,44,45]. In response to high *T*_a_, some species, particularly *C. barabensis, M. meridianus* and *M. unguiculatus*, decreased their metabolic rates to reduce thermal loads, whereas desert rodent species showed no changes in metabolic physiology. One possibility is that desert rodents might have been selected for low RMR, for lowering energy expenditure in the desert environment, but might have less metabolic plasticity when they experience long-term heatwaves, disassociated with a large reduction in gut microbiota diversity. This unmatched relationship between the gut microbiota and host metabolic plasticity in desert species might affect their fitness when they experience heatwaves above the upper critical temperature. However, thermal tolerance across rodent species still needs to be further investigated. Combining microbial and host metabolic data, we demonstrate that the microbial responses to different *T*_a_s seem to be phylogenetically conserved, whereas host metabolic plasticity at different *T*_a_s did not seem to link with the phylogenic relationship.

Altogether, we provide evidence of the relative contributions of phylogeny and environment to both the gut microbial community and rodent metabolic phenotypes on both short-term and evolutionary timescales, by combining field sampling and laboratory manipulations. The branching orders of the gut microbial dendrogram are nearly congruent with the phylogenetic relationships of host species independent of the environment; whereas host metabolic traits are decoupled from phylogeny. Simulated heatwaves lead to a large decrease in core microbes of rodent species and the desert species exhibit decreased metabolic plasticity and more loss in gut microbiota diversity in response to heatwaves. These findings deepen our understanding of the association between microorganisms and their animal hosts, and demonstrate that the divergent variations in gut microbiota across rodent species preserve the phylogenetic signal, but the metabolic physiology implies functional convergence independent of host phylogeny. A major limitation of the experiments for laboratory manipulation was the absence of true negative control and there was a strong temporal autocorrelation in the data set. This limitation was the result of a lack of laboratory-reared wild rodents for all species, to make measures on independent groups. Future studies might seek to further address these issues. Additionally, it would be of importance to take the association between microorganisms and their animal hosts into consideration when predicting species’ responses and population dynamics in the context of global warming and, in particular, frequently occurring heatwaves.

## METHODS

### Species selection

Wild individuals of five rodent species (*C. barabensis, D. sagitta, M. meridianus, M. unguiculatus* and *P. roborovskii*) were live-trapped in the sand dunes of Wuritu (43°7′54′′N, 116°6′56′′E) in Inner Mongolia in July 2019. The laboratory-reared populations of seven species (*C. barabensis, D. sagitta, L. brandtii, M. meridianus, M. unguiculatus, P. roborovskii* and *P. campbelli*) were primarily live-trapped from the desert grassland habitat in Inner Mongolia. These seven species are distributed in three main landscapes: grasslands, desert grassland and desert regions. These rodents were reared in the laboratory at 23 ± 1ºC and under a light cycle of 16L: 8D for at least 6 months. The SDs for these species were supplied and all animal procedures were approved (see Supplementary Data).

### Experimental designs

#### Exp. 1: species differences in the gut microbiota of wild and laboratory-reared rodents

To compare the differences across species and between wild and laboratory-reared animals, we collected fresh fecal samples from wild-living animals (adult, both males and females, *n* = 6–12 per species) and laboratory-reared animals (adult, both males and females, *n* = 8–16 per species; see Supplementary Data).

#### Exp. 2: gut microbial responses to diet changes

To compare species differences and test diet effects on gut microbiota, the 7 laboratory-reared rodent species (adult, *n* = 7–15 per species) were fed with an SD (as the control) for at least 6 months and then fed with the other diet (CD) for another 3 weeks. The diet compositions were detailed and fresh feces were collected (see Supplementary Data).

#### Exp. 3: microbial and host metabolic responses to high *T*_a_

To examine the effects of species and high *T*_a_ on the diversity of gut microbial communities and host metabolic traits, all 7 species (adult, *n* = 8–16 per species) were reared at room temperature (23 ± 1ºC, as the control) and fed with an SD for at least 3 weeks, and then acclimated to high *T*_a_ (32 ± 1ºC) for another 3 weeks. The metabolic traits were measured, and blood and fresh feces were collected (see Supplementary Data).

### RMR measurement

Metabolic trials were conducted for 3 hours via an open-flow respirometry system (TSE LabMaster, Germany). The details are supplied (see Supplementary Data).

### Serum thyroid hormone assays

Serum total T3 and T4 levels were quantified by radioimmunoassay kits (see Supplementary Data).

### Fecal DNA extraction, evaluation and amplification

Total DNA was extracted and purified from fecal samples, and the V3–V4 hypervariable regions were amplified (see Supplementary Data).

### 16S rRNA gene amplicon sequencing and analysis

All sequence analyses were performed using the QIIME software suite, according to the Qiime tutorial (http://qiime.org/) with some modified methods (see Supplementary Data).

### Host phylogenetic inference

The mtDNA sequences of *Cytb* and *COI*, and nDNA of *IRBP* were downloaded from GenBank (Table S6). Bayesian analysis was employed to reconstruct the phylogeny of these species (see Supplementary Data).

### Statistical analysis

The detailed statistical analysis was described (see Supplementary Data).

## Supplementary Material

nwad209_Supplemental_File

## Data Availability

Raw sequence data are available in the NCBI Sequence Read Archive under accession PRJNA989385 and PRJNA992935.
