# Supplementary material for: Phylogenetic signal in gut microbial community rather than in rodent metabolic traits
Source: Natl Sci Rev. 2023 Jul 28;10(10):nwad209. doi: 10.1093/nsr/nwad209 (PMC10625476; doi:10.1093/nsr/nwad209)
Supplement: nwad209_Supplemental_File [file nwad209_supplemental_file.doc]

Supplementary Materials for

# Phylogenetic signal in gut microbial community rather than in rodent metabolic traits

Xue-Ying Zhang^1^†*, Saeid Khakisahneh^1^†, Wei Liu^1^, Xinyi Zhang^3,8^, Weiwei Zhai^3,8^, Jilong Cheng^3^, John R Speakman^4,5,6^* and De-Hua Wang^1,2,7^*

***** Corresponding authors. E-mails: zhangxy@ioz.ac.cn; j.speakman@abdn.ac.uk; dehuawang@sdu.edu.cn

**METHODS**

**Species collection**

The wild individuals of 5 rodent species (*C. barabensis*, *D. sagitta*, *M. meridianus*, *M. unguiculatus* and *P. roborovskii*) were live-trapped in the sand dunes located at Wuritu (43°7′54′′N, 116°6′56′′E) of Inner Mongolia in July 2019. The laboratory-reared populations of 7 species (*C. barabensis*, *D. sagitta*, *L. brandtii*, *M. meridianus*, *M. unguiculatus*, *P. roborovskii* and *P. campbelli*) were primarily live-trapped from the desert grassland habitat in Inner Mongolia of China. These 7 species are distributed in 3 main landscapes: grassland, desert grassland, and desert regions. These rodents were reared in the laboratory at 23±1ºC and under a light cycle of 16L: 8D for at least 6 months. The herbivorous *L. brandtii* was fed with standard rabbit pellets (fiber 12.4%, protein 16.6%, fat 3.9%, calorie 16.23 kJ/g) and other species were fed standard rat pellet chow (fiber 3.5%, protein 21.0%, fat 5.4%, calorie 17.58 kJ/g, Beijing KeAo Bioscience Co.) and water was provided *ad libitum*. All animal procedures were approved under the Animal Care and Use Committee of Institute of Zoology, Chinese Academy of Sciences.

**Experimental designs**

**Exp. 1 Species differences in the gut microbiota of wild and laboratory-reared rodents**

To compare the differences across species and between wild and laboratory-reared animals, we collected the fresh fecal samples from wild-living animals (adult, both males and females, *n* = 6-12 per species) and laboratory-reared animals (adult, both males and females, *n* = 8-16 per species). Each animal was put in a clean cage and fresh fecal samples were placed into sterile tubes, immediately frozen in liquid nitrogen, and subsequently stored at −80°C for DNA extraction and 16S rRNA gene sequencing. The wild animals were released at the place where they were captured after the fecal samples were collected. *L. brandtii* and *P. campbelli* were not included in the wild samples due to low density of population in these areas. Thus, we compared 5 species between wild and laboratory-reared populations.

**Exp. 2 Gut microbial responses to diet changes**

To compare species differences and test diet effects on the gut microbiota, the laboratory-reared 7 rodent species (adult, *n* = 7-15 per species) were fed with a standard diet (SD, as the control) at least for 6 months and then changed to be fed with the other diet (CD) for another 3 weeks. Based on the previous studies, the small mammals exhibited stable metabolic phenotypes after 3-week high-fiber-diet or high-protein-diet acclimations [1, 2]. *L. brandtii* was changed to be fed rat pellets and other species were changed to be fed rabbit pellets. At the end of each diet acclimation, the fresh fecal samples of individuals were collected and stored at −80°C for later DNA extraction and 16S rRNA gene sequencing.

**Exp. 3 Microbial and host metabolic responses to high *T*_a_**

To examine the effects of species and high air temperature (*T*_a_) on the diversity of gut microbial communities, all 7 species (adult, *n* = 8-16 per species) were reared at room temperature (23 ± 1ºC, as the control) and fed with SD at least for 3 weeks, and then were acclimated to high temperature (32 ± 1ºC) for another 3 weeks. The previous studies showed that a 3-week high or low *T*_a_ acclimation was enough for the small mammals to produce stable metabolic phenotypes [3, 4]. The high *T*_a_ was set within the thermoneutral zones (TNZ) of all species. At the end of each *T*_a_ phase, the fresh fecal samples of each individual were collected and stored at −80°C for later DNA extraction and16S rRNA gene sequencing.

We also measured host metabolic traits in different species acclimated at 23 ± 1ºC and 32 ± 1ºC to examine the physiological responses to elevated *T*_a_. During each temperature acclimation, body mass and RMR were measured, and blood was collected from the infraorbital vein for measurement of serum thyroid hormones (T3 and T4).

**RMR measurement**

Metabolic trials were conducted for 3 hours via an open-flow respirometry system (TSE LabMaster, Germany) as previously described [5]. Animals were individually placed in the chamber (2.7 L), which was set at 30 ± 0.5 °C (within TNZ of all species). Fresh air was constantly pumped through 3 chambers (a blank chamber included) and flow rate for *D. sagitta* was 1 L/min, for *M. unguiculatus* and *M. meridianus* was 0.9 L/min and for other species was 0.6 L/min. For each individual the average of 3 consecutive and minimum readings of oxygen consumption was taken as the RMR at least after 1 h acclimation in metabolic chamber. All the metabolic trials were conducted at daytime from 0700 h–1700 h.

**Serum thyroid hormone assays**

Serum total T3 and T4 levels were quantified by radioimmunoassay kits (T3, A01PJB; T4, A02PJB; China Institute of Atomic Energy, Beijing) according to the instructions which have been described before [4, 6]. The intra-assay coefficients of variation (CV) was 2.4% for T3 and 4.3% for T4, and the inter-assay CV was 8.8% for T3 and 7.6% for T4.

**Fecal DNA extraction, evaluation and amplification**

Total DNA was extracted through 2×CTAB (Cetyltrimethyl Ammonium Bromide), Phenol chloroform mixture (phenol: chloroform: isoamyl alcohol = 25:24:1), and was isolated via the spin column from SanPrep Column DNA Gel Extraction Kit (Sangon Biotech, China). For each set of DNA extractions, the negative DNA extraction controls were processed with no fecal pellets (an ampty tube exposed to laboratory air). DNA purity was assessed by absorbance on a Nanodrop 2000 (Thermo Fisher Scientific, Carlsbad, CA, USA) by measuring the A260/A280 ratio.

Using two bacterial 16S rRNA gene amplicon PCR primers (forward primer-341F, CCTACGGGNGGCWGCAG; reverse primer-805R, GACTACHVGGGTATCTAATCC), we amplified the V3–V4 hypervariable regions [6, 7, 8]. The PCR reaction was set up as follows (total 20 μL): template DNA 2 μL, amplicon PCR forward primer (10 μM) 1 μL, amplicon PCR reverse primer (10 μM) 1 μL, and 2×Taq PCR MasterMix 16 μL. PCR was performed in the same thermal cycler (SimpliAmp, ABI) using the following program: 1 cycle of denaturing at 94°C for 3 min, 6 cycles of denaturing at 94°C for 20 s, annealing at 55°C for 30 s, elongation at 72°C for 30 s, then followed by 30 cycles of denaturing at 94°C for 15 s, annealing at 68°C for 15 s, elongation at 72°C for 20 s, and a final extension at 72°C for 5 min. PCR products were detected using 1% agarose gel (w/v) electrophoresis and stained with ethidium bromide and visualized under UV light. And then the PCR products were purified using SanPrep Column DNA Gel Extraction Kit (GE0101-200, TsingKe, Beijing, China) according to the manufacturer’s instructions.

**16S rRNA gene amplicon sequencing and analysis**

The sequencing was done on an Illumina HiSeq 2500. The 16S sequence paired-end data set was joined and quality was filtered using the FLASH method. All sequence analyses were performed using QIIME (version 1.9.1) software suite, according to the Qiime tutorial (http://qiime.org/) with some modified methods. The chimeric sequences were removed using usearch61 with de novo models. Sequences were clustered against the 2013 Greengenes (13_5 release) ribosomal database's 97% reference dataset. Sequences which did not match any entries in this reference were subsequently clustered into de novo OTUs at 97% similarity with UCLUST. Taxonomy was assigned to all OTUs using the RDP classifier within QIIME and the Greengenes reference data set. The rarefaction and rank abundance curves were calculated from OTU tables using alpha diversity and rank abundance scripts within the QIIME pipeline. The hierarchical clustering based on population profiles of most common and abundant taxa was performed through UPGMA clustering (Unweighted Pair Group Method with Arithmetic Mean, also known as average linkage) on the distance matrix of OTU abundance and a Newick formatted tree was obtained using the QIIME package.

**Host phylogenetic inference**

We downloaded mtDNA sequences of *Cytb* and *COI* for these 7 species, and nDNA of *IRBP* for 6 species except *P. campbelli* from GenBank (accession numbers were given in Table S5). Bayesian analysis was employed to reconstruct the phylogeny of these species. Jmodeltest 2.1.7 was run independently to select the best fit model of base substitution for each gene partition based on the Bayesian information criterion (BIC) [9], and MrBayes v.3.2.7 was used to estimate the tree topologies [10]. Two parallel runs, 1 cold and 3 heated chain of Markov Chain Monte Carlo (MCMC) analyses were performed for 20 million generations, with trees sampled every 1,000 generations to reduce autocorrelation (SD < 0.01). The first 25% of the Markov chain samples (*n* = 20,000) were discarded as burn-in, and the remaining samples were used to generate majority rule consensus trees. Final trees were arranged and formatted using FigTree v1.4.2 (available at http://tree.bio.ed.ac.uk/software/fig-tree/). The phylogenetic trees were also checked through the timescale of life (http://www.timetree.org/).

With multiple phenotypic values (e.g. gut microbial profiles or physiological status), we inferred the phylogenetic relationship among these species using a collection of phenotypic traits. Since different phenotypic traits can be measured in different units, we first rescaled each trait across species and subsequently calculated the Euclidian distance between pairs of species. Using this distance matrix between all pairs of species, we inferred the dendrogram relationship using the neighbor-joining algorithm. In addition, we also analyzed the correlation between the distance matrix of phenotypic divergence and the phylogenetic distance inferred using nucleotide sequences. The heatmaps of rescaled phenotypic traits were drawn via R-package pheatmap, and the phylogenetic relationship comparisons were plotted via R-package phytools. The phylogenetic tree across the genera of bacteria was build up through PhyloPlus software according to taxonomy ID in NCBI.

**Statistical analysis**

Beta diversity of microbial communities was indicated by PCoA based on Bray-Curtis distances using evenly sampled OTU abundances and the statistics were detected with multivariate permutation tests using the nonparametric method “ANOSIM” test included in the R “vegan” package of the QIIME-incorporated version. The similarities in microbial structures across different host species were investigated via the beta_diversity_through_plots.py script in QIIME using 24,000 sequences from each of the pooled microbial communities of each species at 23^o^C. The Venn graphs showing core microbes of 7 species were done through TBtool software. The LEfSe method was used to show the biomarkers and the differences in microbial communities were set at an LDA score threshold of 2. We calculated the Robinson-Foulds (RF) distance by using R package TreeDist, and used permutations to assess the significance of RF distances using phytools (<https://rdrr.io/cran/phytools/man/cospeciation.html>) to compare the bipartitions across trees [11, 12, 13]. Pearson correlation coefficients and Mantel tests by R package vegan were performed to assess the correlation for β diversity distances and all metabolic differences with phylogenetic divergence. We tested for the phylogenetic signal (using https://rdrr.io/cran/RPANDA/man/phylosignal_network.html) in rodent species and bacteria (Top 30 genera) interactions using the R-package GUniFrac and Mantel tests based on Pearson correlation, and the significance of every correlation was evaluated by bootstrapping with 10,000 permutations [14, 15]. To account for phylogenetic relationships between rodent species, phylogenetic generalized linear models (PGLS) were used for the statistical analyses [16]. Caper package was used for PGLS to take into account the phylogenetic non-independence between species. The branch length transformation lambada was optimized using maximum likelihood. One-way ANOVA and Tukey post hoc tests were further used to compare differences between species for the parameters without phylogenetic relationship. The Pearson correlation analyses between specific OTUs and metabolic parameters were evaluated with 1000 permutations and the level of statistical significance was set at *P* < 0.05 (False Discovery Rate (FDR)-corrected *P* value) to exclude false positives.

To remove the effects of body mass of different species, RMR were corrected by the 0.67 power of body mass as proposed previously for rodents [17, 18]. The body mass–corrected RMR, thyroid hormones and the relative abundance of bacteria among rodent species were analyzed by PGLS and one-way ANOVA, and significant species differences were further evaluated using Tukey post hoc tests. The differences in body mass–corrected RMR, thyroid hormones and bacteria abundance between 23ºC and 32ºC were assessed by a paired sample *t*-test for each species. The SPSS 17.0 software was used for statistical analyses. Results were presented as mean ± SEM, and *P* < 0.05 was considered to be statistically significant.

**References**

1. Zhao ZJ, Wang DH. Effects of diet quality on energy budgets and thermogenesis in Brandt's voles. *Comp Biochem Physiol A*. 2007; **148**: 168-177.
2. Lou MF, Zhang XY, Fu RS, Wang DH. Effects of dietary fiber content on energetics in nonreproductive and reproductive Brandt’s voles (*Lasiopodomys brandtii*). *Can J Zool*. 2015; **93**: 251-258.
3. Zhang XY, Wang DH. Energy metabolism, thermogenesis and body mass regulation in Brandt‘s voles (*Lasiopodomys brandtii*) during cold acclimation and rewarming. *Horm Behav*. 2006; **50**: 61-69.
4. Khakisahneh S, Zhang XY, Nouri Z, Hao SY, Chi QS, Wang DH. Thyroid hormones mediate metabolic rate and oxidative, anti-oxidative balance at different temperatures in Mongolian gerbils (*Meriones unguiculatus*). *Comp Biochem Physiol C Toxicol Pharmacol*. 2019; **216**: 101-109.
5. Chi QS, Li XJ, Wang DH. 2-Deoxy-D-glucose, not mercaptoacetate, induces a reversible reduction of body temperature in male desert hamsters (*Phodopus roborovskii*). *J Therm Biol*. 2018; **71**: 189-194.
6. Khakisahneh S, Zhang XY, Nouri Z, Wang DH. Gut microbiota and host thermoregulation in response to ambient temperature fluctuations. *mSystems* 2020; **5**: e00514-20.
7. Zhang XY, Sukhchuluun G, Bo TB, Chi QS, Yang JJ, Chen B, Zhang L, Wang DH. Huddling remodels gut microbiota to reduce energy requirements in a small mammal species during cold exposure. *Microbiome* 2018; **6**: 103.
8. Doherty FD, O'Mahony SM, Peterson VL, O'Sullivan O, Crispie F, Cotter PD, Wigmore P, King MV, Cryan JF, Fone KCF. Post-weaning social isolation of rats leads to long-term disruption of the gut microbiota-immune-brain axis. *Brain Behav Immun.* 2018; **68**: 261-273.
9. Darriba D, Taboada GL, Doallo R, Posada D. jModelTest 2: more models, new heuristics and parallel computing. *Nature Methods*. 2012; **9**: 772-772.
10. Ronquist F, Teslenko M, van der Mark P, Ayres DL, Darling A, Hohna S, Larget B, Liu L, Suchard MA, Huelsenbeck JP. MrBayes 3.2: Efficient Bayesian Phylogenetic Inference and Model Choice Across a Large Model Space. *Syst. Biol.* 2012; **61**: 539-542.
11. Robinson DF, Foulds LR. Comparison of phylogenetic trees. *Math Biosci*. 1981; **53**: 131–147.
12. Smith MR. TreeDist: Distances between Phylogenetic Trees. R package version 2.6.1. 2020; doi: 10.5281/zenodo.3528124.
13. Revell LJ. phytools: An R package for phylogenetic comparative biology (and other things). *Methods Ecol Evol*. 2012; **3**, 217-223.
14. Morlon H, Lewitus E, Condamine FL, Manceau M, Clavel J, Drury J. RPANDA: an R package for macroevolutionary analyses on phylogenetic trees. *Methods Ecol Evol*. 2016; **7**: 589-597.
15. Perez-Lamarque B, Maliet O, Pichon B, Selosse MA, Martos F, Morlon H. Do closely related species interact with similar partners? Testing for phylogenetic signal in interaction networks. *bioRxiv*. 2022. doi:10.1101/2021.08.30.458192.
16. Freckleton RP, Harvey PH, Pagel M. Phylogenetic analysis and comparative data: A test and review of evidence. *American Naturalist*. 2002; **160**:712-726.
17. Heusner AA. Size and power in mammals. *J Exp Biol*. 1991; **160**: 25-54.
18. White CR, Seymour ES. Allometric scaling of mammalian metabolism. *J Exp Biol*. 2005; **208**:1611-9.


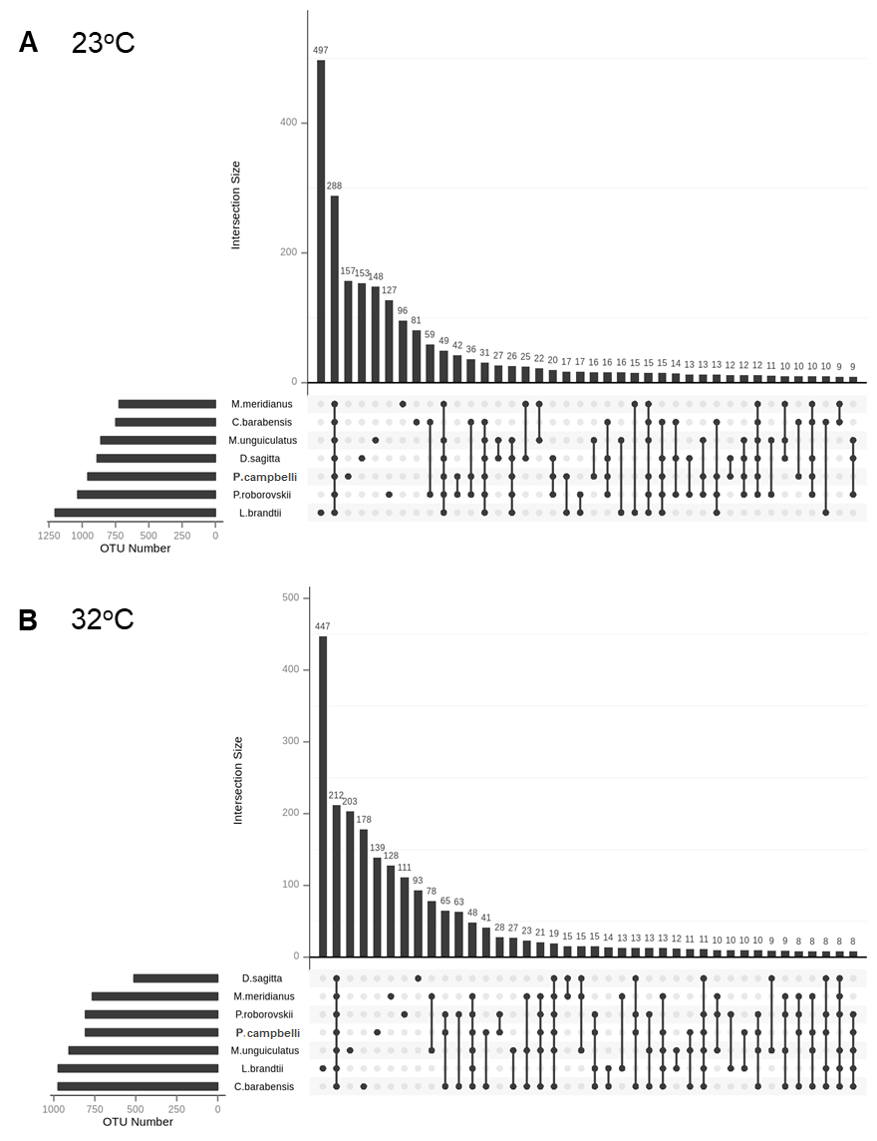


**Figure S1** **The core OTUs present in across 90% of samples in each rodent species.**

(*A*) At 23^o^C. (*B*) At 32^o^C. Left is the OTU number in each species and right is the intersection size for different assemble groups.


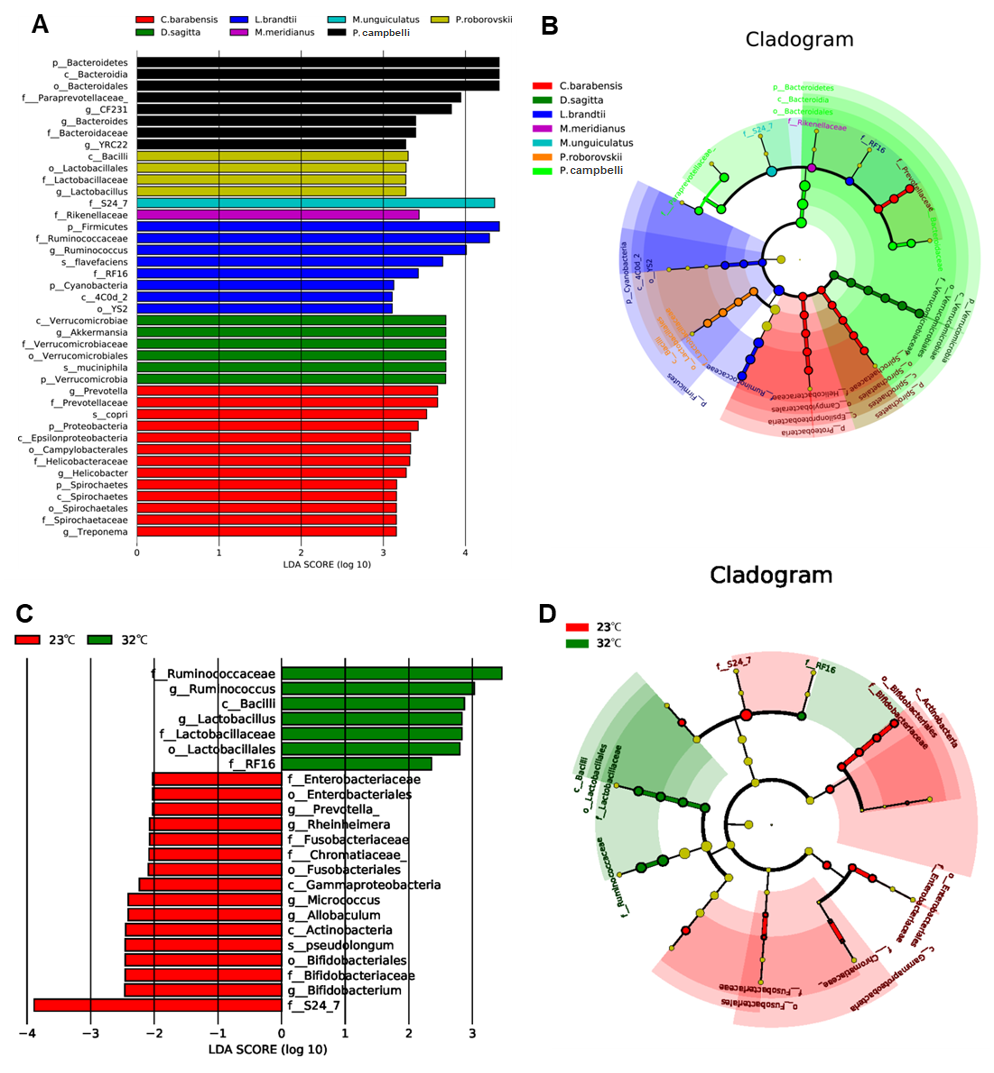


**Figure S2** **Biomarkers of bacterial taxa in each animal species and at different temperatures.**

(*A, C*) Differential bacterial taxa selected by LEfSe analysis with LDA >2 in fecal microbial community of 7 rodent species. (*B, D*) Cladogram representing taxa enriched in fecal microbial community at 23^o^C and 32^o^C, detected by the LEfSe tool with LDA >2.


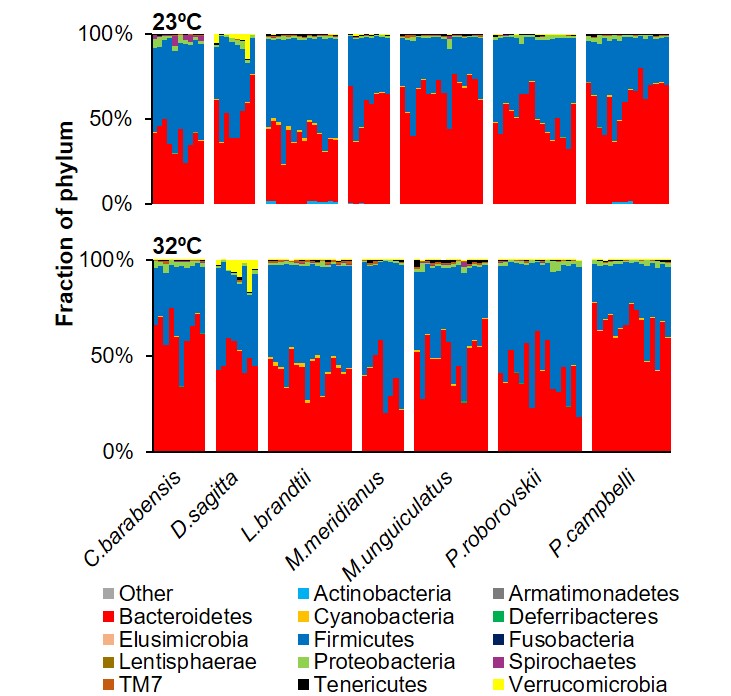


**Figure S3 The fraction of phylum of microbial community in fecal samples across 7 rodent species at 23^o^C and 32^o^C.**


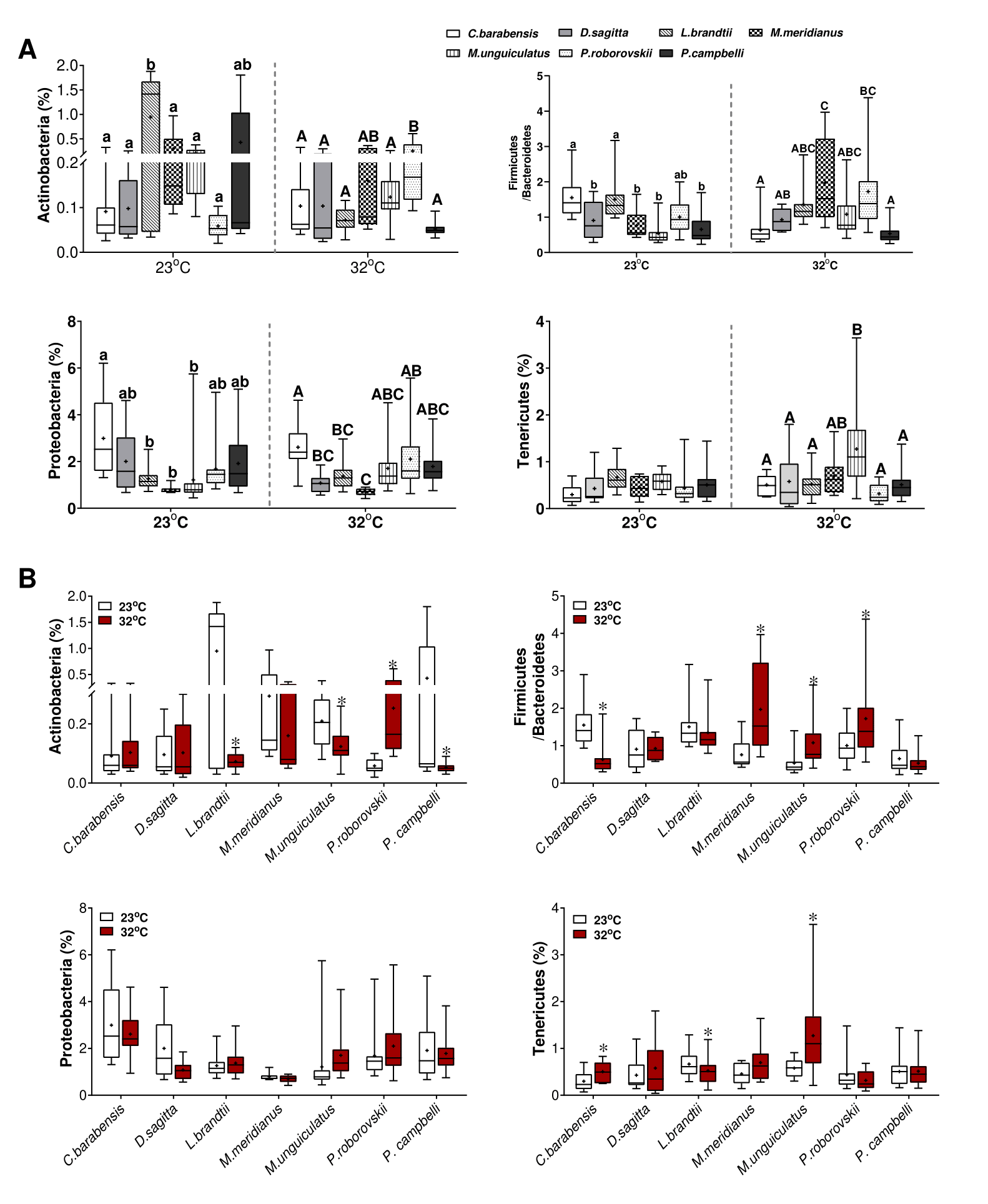


**Figure S4** **Relative abundance of different bacteria at the phylum level.**

“+”indicates the mean of data. Different small and capital letters above box plots indicate significant differences among species at 23^o^C and 32^o^C, respectively (*P* < 0.05). **P* < 0.05, 32^o^C vs 23^o^C.


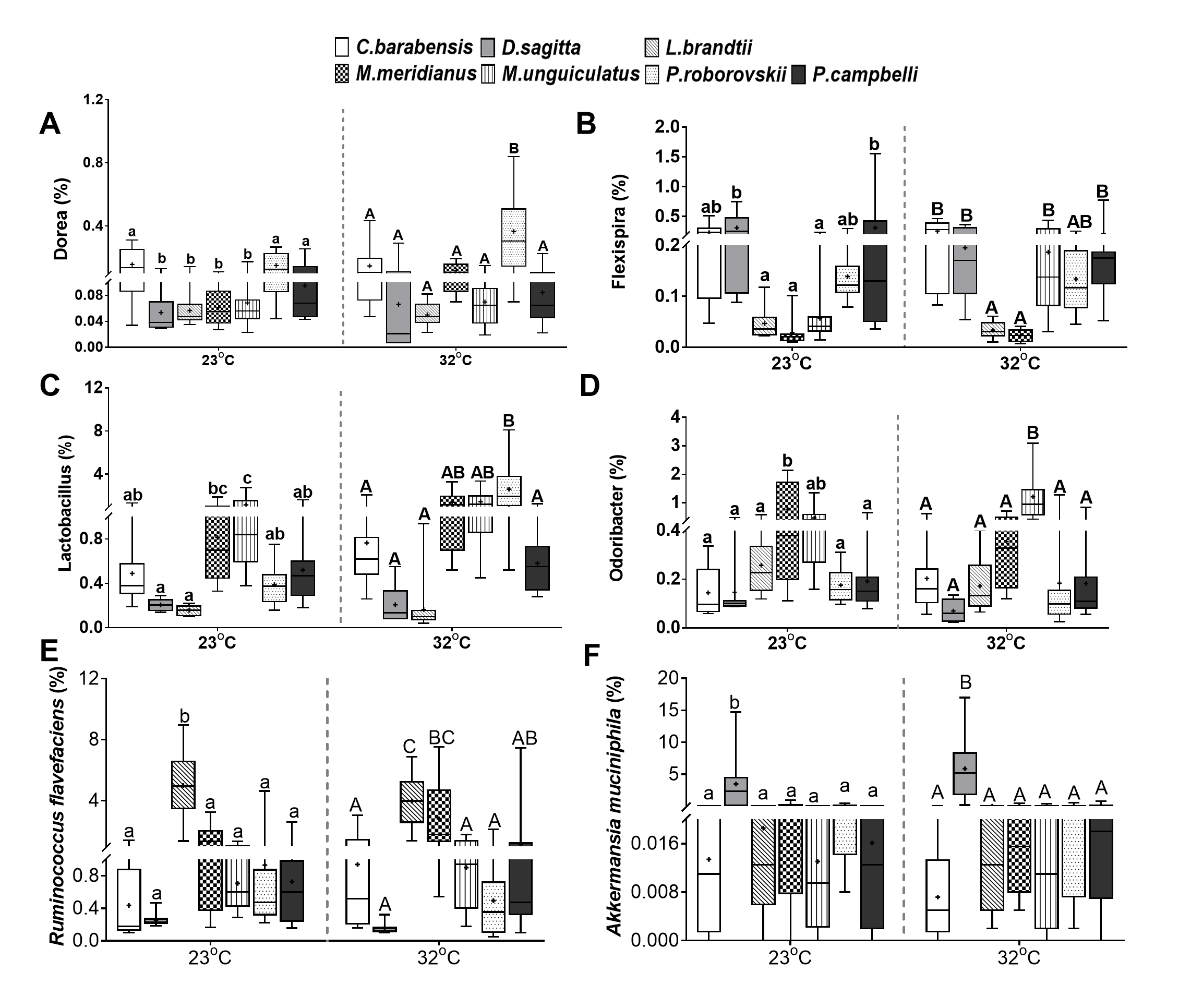


**Figure S5** **Variations in the relative abundance of bacteria at the genus and species levels among host species at 23^o^C and 32^o^C.**

Different small and capital letters above box plots indicate significant differences among species at 23^o^C and 32^o^C, respectively (*P* < 0.05).

**
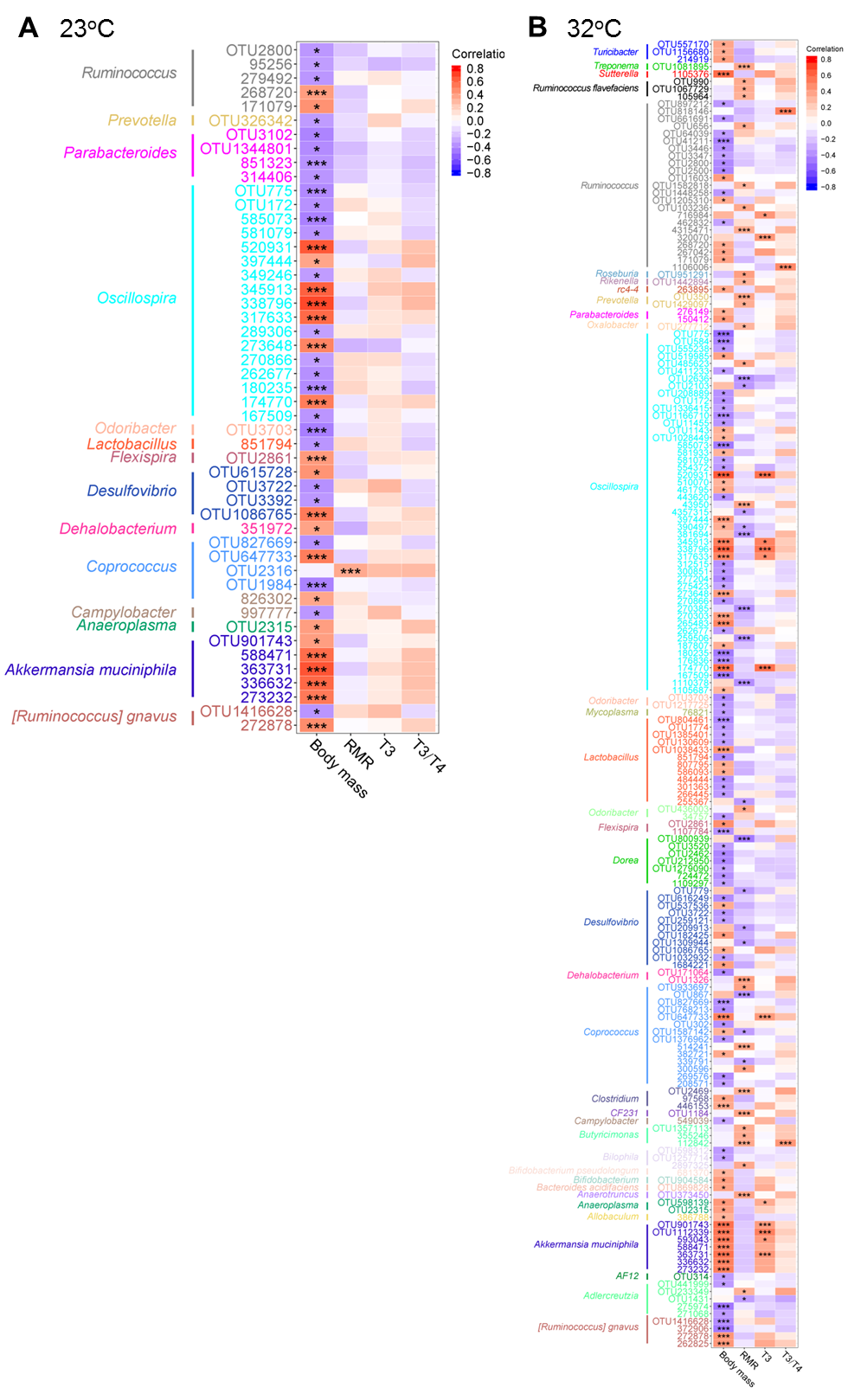
**

**Figure S6 The correlation between host metabolic physiology and specific OTUs at different *T*_a_s.**

(A, B) The heatmaps showed positive or negative correlations between host physiological traits and specific OTUs at 23^o^C and 32^o^C. * *P* < 0.05, *** *P* < 0.001 (False Discovery Rate (FDR)-corrected *P* value).

**Table S1 The statistics for testing phylogenetic signal with an interaction network between hosts and the genus (top 30)** of bacteria with associated phylogenetic trees.

| Factors | Tree a | | | |  | Tree b | | |
| --- | --- | --- | --- | --- | --- | --- | --- | --- |
|  | Mantel_corr | | pvalue_upper | pvalue_lower |  | Mantel_corr | pvalue_upper | pvalue_lower |
| **Weighted** |  |  | |  |  |  |  |  |
| Step 1 |  |  | |  |  |  |  |  |
| Wild_lab | 0.850 | 0.032 | | 0.986 |  | 0.151 | 0.021 | 0.979 |
| Diets | 0.736 | 0.008 | | 0.992 |  | 0.178 | 0.020 | 0.980 |
| *T*_a_ | 0.481 | 0.037 | | 0.963 |  | 0.127 | 0.062 | 0.938 |
| Step 2 |  |  | |  |  |  |  |  |
| Wild_lab | 0.850 | 0.013 | | 0.989 |  | 0.151 | 0.100 | 0.902 |
| Diets | 0.736 | 0.016 | | 0.986 |  | 0.178 | 0.009 | 0.993 |
| *T*_a_ | 0.481 | 0.378 | | 0.624 |  | 0.127 | 0.039 | 0.963 |
| **Unweighted** |  |  | |  |  |  |  |  |
| Step 1 |  |  | |  |  |  |  |  |
| Wild_lab | 0.714 | 0.016 | | 1.000 |  | 0.065 | 0.093 | 0.907 |
| Diets | 0.591 | 0.021 | | 0.980 |  | 0.055 | 0.089 | 0.911 |
| *T*_a_ | 0.494 | 0.039 | | 0.962 |  | -0.015 | 0.602 | 0.398 |
| Step 2 |  |  | |  |  |  |  |  |
| Wild_lab | 0.714 | 0.141 | | 0.861 |  | 0.065 | 0.132 | 0.870 |
| Diets | 0.591 | 0.744 | | 0.258 |  | 0.055 | 0.188 | 0.814 |
| *T*_a_ | 0.494 | 0.388 | | 0.614 |  | -0.015 | 0.585 | 0.417 |

Tree_a = tree.host, tree_b = tree.genus, method = “GUniFrac”, correlation = “Pearson”, nperm =10000 for step 1 and 1000 for step 2, permutation = “nbpartners” for step 2.

pvalue_upper < 0.05, indicating a significant phylogenetic signal in guild a (or b).

pvalue_lower < 0.05, indicating a significant anti-phylogenetic signal in guild a (or b).

**Table S2 The lambda and *P*-value of the phylogenetic generalized linear models (PGLS) for the α diversity of gut microbiota community.**

| Parameters | 23^o^C | |  | 32^o^C | |
| --- | --- | --- | --- | --- | --- |
|  | Lambda | *P*-value |  | Lambda | *P*-value |
| Chao1 | 0 | 2.20🞨10^-16^ |  | 0 | 5.27🞨10^-16^ |
| Observed OTUs | 0 | 9.66🞨10^-16^ |  | 0 | 2.08🞨10^-10^ |
| Shannon index | 0 | 3.68🞨10^-13^ |  | 0 | 8.94🞨10^-12^ |
| Simpson index | 0 | 2.63🞨10^-4^ |  | 0 | 8.59🞨10^-5^ |
| PD whole tree | 0 | 2.20🞨10^-16^ |  | 0 | 2.20🞨10^-16^ |

Lambda = 0, representing no phylogenetic relationship.

**Table S3 Alpha diversity including Chao1, Observed OTUs, Shannon Index, Simpson Index and PD whole tree in 7 species at 23ºC and 32ºC.**

|  | Chao1 | Observed OTUs | Shannon index | Simpson index | PD whole tree |
| --- | --- | --- | --- | --- | --- |
| At 23ºC |  |  |  |  |  |
| *C. barabensis* | 13776±332.14^ab^ | 4403±74.27^a^ | 9.37±0.07^b^ | 0.99±0.001^b^ | 220.60±3.22^ab^ |
| *D. sagitta* | 14387±310.05^bc^ | 4463±82.35^a^ | 8.83±0.21^a^ | 0.97±0.01^a^ | 222.46±3.08^ab^ |
| *L. brandtii* | 15484±343.23 ^d^ | 4789±72.00^b^ | 9.88±0.04^c^ | 0.99±0.001^b^ | 254.16±3.06 ^c^ |
| *M. meridianus* | 12916±280.92 ^a^ | 4462±102.91^a^ | 9.38±0.08^b^ | 0.99±0.001^b^ | 219.33±3.73 ^a^ |
| *M. unguiculatus* | 15061±229.63^cd^ | 4841±51.55^b^ | 9.53±0.05^bc^ | 0.99±0.001^b^ | 231.19±1.92 ^b^ |
| *P. roborovskii* | 18015±236.45 ^e^ | 5242±48.36^c^ | 9.93±0.04^d^ | 0.99±0.001^b^ | 253.48±2.14 ^c^ |
| *P. campbelli* | 17830±316.13 ^e^ | 5236±76.32^c^ | 9.70±0.09^bcd^ | 0.99±0.001^b^ | 252.84±2.10 ^c^ |
| At 32ºC |  |  |  |  |  |
| *C. barabensis* | 12853±249.28^BC^ | 4244±79.25^AB^ | 9.18±0.01^AB^ | 0.99±0.001^B^ | 211.39±2.82^B^ |
| *D. sagitta* | 9718±217.87^A^ | 34794±59.92^A^ | 8.37±0.11^A^ | 0.98±0.001^A^ | 181.88±2.81^AC^ |
| *L. brandtii* | 13188±203.97^C^ | 4323±39.02^C^ | 9.52±0.04^C^ | 0.99±0.001^B^ | 233.20±1.67^D^ |
| *M. meridianus* | 11794±360.75^B^ | 3956±99.23^BC^ | 9.13±0.08^BC^ | 0.99±0.001^B^ | 203.08±4.47^B^ |
| *M. unguiculatus* | 13606±406.93^C^ | 4348±106.87^C^ | 9.37±0.07^BC^ | 0.99±0.001^B^ | 217.68±3.57^C^ |
| *P. roborovskii* | 13708±273.04^C^ | 4408±71.44^C^ | 9.44±0.08^C^ | 0.99±0.001^B^ | 219.76±2.59^C^ |
| *P. campbelli* | 13369±165.00^C^ | 4252±40.79^BC^ | 9.04±0.05^B^ | 0.98±0.001^AB^ | 216.33±2.54^C^ |
| *P*_species_ | <0.001 | <0.001 | <0.001 | <0.001 | <0.001 |
| *P*_Ta_ | <0.001 | <0.001 | <0.001 | 0.554 | <0.001 |
| *P*_species×Ta_ | <0.001 | <0.001 | 0.031 | 0.479 | <0.001 |

Data are mean ± SEM. Different small and capital superscript letters indicate significant differences among species at 23^o^C and 32^o^C, respectively (*P* < 0.05).

**Table S4 The variations in specific bacteria in response to 32ºC.**

| **Phylum** | **Order** | **Family** | **Genus** | **Species** | **32ºC vs 23ºC** | ***P*** |
| --- | --- | --- | --- | --- | --- | --- |
| Actinobacteria | Actinomycetales | Corynebacteriaceae | *Corynebacterium* | *C. kroppenstedtii* | Decrease | 0.02 |
|  |  | Sanguibacteraceae | *Sanguibacter* | *-* | Increase | 0.006 |
|  | Sanguibacteraceae | Bifidobacteriaceae | *Bifidobacterium* | *B. pseudolongum* | Increase | <0.001 |
| Bacteroidetes | Bacteroidales | Porphyromonadaceae | *Dysgonomonas* |  | Decrease | 0.003 |
|  | Flavobacteriales | Flavobacteriaceae | *Flavobacterium* | *-* | Increase | 0.022 |
| Firmicutes | Bacillales | Staphylococcaceae | *Staphylococcus* | *-* | Decrease | 0.022 |
|  | Lactobacillales | Aerococcaceae | *Facklamia* | *-* | Decrease | 0.025 |
|  |  | Lactobacillaceae | *Lactobacillus* | *-* | Decrease | <0.001 |
| Proteobacteria | Rhizobiales | Hyphomicrobiaceae | *Devosia* | *-* | Increase | 0.031 |
|  |  | Methylobacteriaceae | *Methylobacterium* | *-* | Increase | 0.039 |
|  | Alteromonadales | [Chromatiaceae] | *Rheinheimera* | *-* | Increase | 0.007 |
|  | Enterobacteriales | Enterobacteriaceae | *Citrobacter* | *-* | Increase | 0.026 |
|  |  |  | *Erwinia* | *-* | Increase | 0.006 |
|  | Pseudomonadales | Moraxellaceae | *Enhydrobacter* | *-* | Decrease | 0.024 |
|  |  | Pseudomonadaceae | *Pseudomonas* | *-* | Increase | 0.022 |
|  | Campylobacterales | Helicobacteraceae | *Flexispira* | *F. rappini* | Increase | 0.018 |
|  | Pseudomonadales | Moraxellaceae | *Acinetobacter* | *A. johnsonii* | Increase | 0.049 |

Significant differences between temperatures were set at *P* < 0.05.

**Table S5 The lambda and *P*-value of the phylogenetic generalized linear models (PGLS)** for host metabolic traits.

| Parameters | 23^o^C | |  | 32^o^C | |
| --- | --- | --- | --- | --- | --- |
|  | Lambda | *P*-value |  | Lambda | *P*-value |
| Body mass | 0 | 2.20🞨10^-16^ |  | 0 | 2.20🞨10^-16^ |
| RMR | 0 | 2.88🞨10^-3^ |  | 0 | 8.12🞨10^-6^ |
| T3 | 0 | 0.022 |  | 0 | 6.50🞨10^-10^ |
| T3/T4 | 0 | 0.013 |  | 0 | 3.50🞨10^-6^ |

Lambda = 0, representing no phylogenetic relationship.

RMR, resting metabolic rate; T3, 3,5,3’-tri-iodothyronine; T4, thyroxine.

**Table S6 The accession numbers for the 3 genes (*Cytb*, *COI* and *IRBP*) in 7 species from GenBank.**

|  | *Cytb* | *COI* | *IRBP* |
| --- | --- | --- | --- |
| *Cricetulus barabensis* | MH340484 | KJ466858 | MG685585 |
| *Dipus sagitta* | KX399737 | KX399597 | MF535798 |
| *Lasiopodomys brandtii* | GQ352472 | KP190334 | JF906134 |
| *Meriones meridianus* | AJ851268 | KT763038 | JQ713186 |
| *Meriones unguiculatus* | AF119264 | KJ466779 | FN984755 |
| *Phodopus roborovskii* | GU797445 | KJ466764 | KJ813156 |
| *Phodopus campbelli* | KY754112 | JF444392 | KC953439 |
| *Ochotona princeps* | AY292716 | KY024832 | AY057832 |
